# Advances in closed-loop deep brain stimulation devices

**DOI:** 10.1186/s12984-017-0295-1

**Published:** 2017-08-11

**Authors:** Mahboubeh Parastarfeizabadi, Abbas Z. Kouzani

**Affiliations:** School of Engineering, Deakin University, Waurn Ponds, VIC 3216 Australia

**Keywords:** Deep rain simulation, Closed-loop control, Biomarker, Sensor, Signal conditioning, Stimulator

## Abstract

**Background:**

Millions of patients around the world are affected by neurological and psychiatric disorders. Deep brain stimulation (DBS) is a device-based therapy that could have fewer side-effects and higher efficiencies in drug-resistant patients compared to other therapeutic options such as pharmacological approaches. Thus far, several efforts have been made to incorporate a feedback loop into DBS devices to make them operate in a closed-loop manner.

**Methods:**

This paper presents a comprehensive investigation into the existing research-based and commercial closed-loop DBS devices. It describes a brief history of closed-loop DBS techniques, biomarkers and algorithms used for closing the feedback loop, components of the current research-based and commercial closed-loop DBS devices, and advancements and challenges in this field of research. This review also includes a comparison of the closed-loop DBS devices and provides the future directions of this area of research.

**Results:**

Although we are in the early stages of the closed-loop DBS approach, there have been fruitful efforts in design and development of closed-loop DBS devices. To date, only one commercial closed-loop DBS device has been manufactured. However, this system does not have an intelligent and patient dependent control algorithm. A closed-loop DBS device requires a control algorithm to learn and optimize the stimulation parameters according to the brain clinical state.

**Conclusions:**

The promising clinical effects of open-loop DBS have been demonstrated, indicating DBS as a pioneer technology and treatment option to serve neurological patients. However, like other commercial devices, DBS needs to be automated and modernized.

## Background

**Fig. 1:**
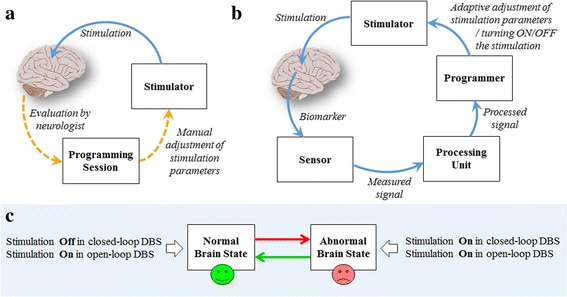
Overview of open-loop DBS (**a**) versus closed-loop DBS (**b**). In open-loop DBS, a neurologist manually adjusts the stimulation parameters every 3–12 months after DBS implantation. On the other hand, in closed-loop DBS, programming of the stimulation parameters is performed automatically based on the measured biomarker. **c** Demonstration of two different brain states and the action of open-loop and closed-loop DBS. When the brain enters a specific state, it remains in that state for a short or long time. Closed-loop DBS gets deactivated when the brain enters the normal state. Open-loop DBS continues the stimulation regardless of the brain state

Deep Brain Stimulation (DBS) can be classified into open-loop (also known as conventional) and closed-loop (also known as adaptive) paradigms. Closed-loop DBS employs a sensor to record a signal linked to symptoms while open-loop DBS does not use a sensor for recording the brain condition; therefore, stimulation parameters including duration, amplitude, and frequency of the pulse train remain constant in open-loop DBS regardless of fluctuations in the disease state. The recorded signal is known as a biomarker and can have varying nature, e.g. bioelectric, physiologic, biochemical, etc. In the open-loop DBS, a specialist tracks the patient’s clinical state and manually programs the device in a trial-and-error based manner. Adjustments of stimulation parameters are not conducted in real-time based on the ongoing neurophysiological variations in the brain; therefore, adverse effects on the patient may be induced due to the brain overstimulation. On the other hand, in the closed-loop DBS, the stimulation pulses are delivered when the brain is in an abnormal state, or they are automatically and dynamically adjusted based on the variations in the recorded signal over the time. Figure 1 compares open-loop and closed-loop DBS and illustrates how they act in different brain states.

Although the conventional DBS is a successful therapy, the closed-loop DBS is potentially capable of further and more efficient improvements in neurological diseases. A systematic review of the clinical literature by Hamani et al. [1] stated that adjusting the stimulation parameters of DBS devices could reduce or abolish adverse effects reported in 142 (19%) of 737 Parkinson’s disease (PD) patients treated with subthalamic nucleus (STN) DBS. In addition, Rosin et al. [2] demonstrated the superior function of closed-loop DBS, which automatically adjusts the stimulation parameters, to alleviate PD symptoms. Moreover, Little et al. [3] indicated that motor scores in eight PD patients improved by 50% (blinded) and 66% (unblinded) during closed-loop DBS, which were 27% (*p* = 0.005) to 29% (*p* = 0.03) higher than that of open-loop DBS. Besides these therapeutic benefits, they reported 56% reduction in stimulation time, as well as a decrease in the energy requirement of the closed-loop DBS compared to open-loop DBS. Therefore, patients may also benefit from fewer surgeries for replacement of the neurostimulator battery as a result of less power consumption in non-continuous stimulations [3]. Little et al. [3] and Wu et al. [4] reported that in order to obtain similar results from open-loop and closed-loop DBS, 44% less electrical stimulation is required using closed-loop DBS, which means higher efficiency, fewer surgery numbers, lower power consumption, and longer battery lifespan.

Although DBS is a successful therapy, its operation mechanism is mainly uncertain. Hess et al. [5] explained how the temporal pattern of stimulations might have key information for clarification of the DBS mechanism. A recent short review [6] on the physiological mechanism of DBS suggests the “disruption hypothesis” in which abnormal information is prevented from flowing into the stimulation site as a result of DBS dissociation effect on input and output signals. However, it is still under debate and remains to be confirmed by more pre-clinical research. Another review by Herrington et al. [7] accounts several non-exclusive mechanisms for DBS that depend on the condition being treated and the stimulation target. Despite the existence of different theories on the DBS mechanism, there are still questions in regard to the closed-loop DBS. Does adaptive control of DBS alter the DBS mechanism? If yes, how does it alter the DBS mechanism? These questions deserve consideration in the future experimental studies.

This paper presents a comprehensive review of portable closed-loop DBS devices. While there exists a number of excellent reviews on closed-loop DBS systems [8–16], this work differs from the existing works as described in the following. Among the published reviews, ref. [8] mainly highlights the applications of closed-loop DBS in the rehabilitation of movement disorders. Ref. [12] mainly describes the benefits of closed-loop DBS which using local field potentials (LFPs) as the feedback biomarker. Ref. [13] mainly reviews DBS (both open-loop and closed-loop) in terms of neurological aspects and clinical benefits. Ref. [9] indicates the available biomarkers for closing the feedback loop, and gives control strategies for manipulating measured signals relating to PD patient clinical state. Ref. [10] concentrates on emerging techniques in DBS including new electrode design, new stimulation patterns, and novel targeting techniques. Ref [16] has mainly focused on selection of biomarker and its benefits and problems. Ref. [14] introduces adaptive DBS, and outlines some technological advances in DBS including stimulation type and patterns, energy harvesting, and methods for increasing life quality of patients. Similarly, ref. [15] reviews some technological advancement such as surgical targeting, DBS parameters programming, and electrode design. On the other hand, ref. [11] highlights a range of issues associated with closed-loop DBS including biomarker sensing and processing, DBS parameters programming, control algorithm, wireless telemetry, and device size and power consumption.

This paper, on the other hand, provides a comprehensive review of closed-loop DBS devices, and covers a wider range of issues and advancements associated with such devices including: (i) biomarker selection, (ii) DBS parameters programming, (iii) stimulation type and pattern, (iv) control algorithms, (v) concurrent stimulation and recording, (vi) portability, (vii) battery-less technique, (viii) user-friendly interface, and (x) remote monitoring and wireless telemetry. The paper combines the key features of the current reviews going beyond devices that are used for specific disorders or biomarkers. It covers closed-loop DBS devices reported in the latest research publications not included in the existing reviews. The paper gives a brief history of closed-loop DBS. Next, it discusses different biomarkers for closing the feedback loop. Then, it reviews the algorithms developed for controlling stimulation parameters. After that, it highlights the current challenges and limitations for implementing closed-loop DBS. Also, it reviews the technological developments in closed-loop DBS. Then, it describes commercial closed-loop DBS systems. After that, it compares research-based closed-loop DBS devices highlighting future design expectations, and giving future directions and recommendations on closed-loop DBS devices.

## Brief history of closed-loop DBS

The review of scientific literature reveals that the initial use of closed-loop against open-loop DBS goes back to early 2000 [17] when an ultra-short-term closed-loop neurostimulator device was introduced being capable of stimulation by detection of seizures. This initial pilot study led to optimistic findings which were confirmed by other studies during 2002–2005. These studies [18, 19] concentrated on an external responsive neurostimulator (RNS) system capable of detecting seizures, delivering stimulations in a semi-closed-loop manner, and storing electrocorticogram (ECoG) potentials. These studies had promising consequences on seizure activity of 11 out of 27 patients. However, due to the low specificity of seizure detection of the RNS [20], it was not entirely considered as an adaptive DBS triggered by seizures [21].

Following the use of a semi-closed-loop DBS device on epileptic patients, the idea of dynamically controlling DBS became a target for many researchers. However, implementation of closed-loop DBS was postponed due to the difficulties associated with technical and computational aspects, and the reliability of selected biomarkers. Selection of biomarkers and control algorithm for epilepsy could be less complicated than that for other neurological disorders (because of a distinguishable shape of the seizures activities compared to the non-seizure neural signal). Thus, lack of reliable biomarkers for most of neurological disorders postponed the development of a closed-loop DBS device for such disorders. Later, various investigations were initiated to address the problems associated with the use of closed-loop DBS for several neurological disorders. These attempts have been performed towards developments of closed-loop DBS devices, which are discussed in detail in the later sections of this review.

## Biomarkers for closing of the feedback loop

**Fig. 2:**
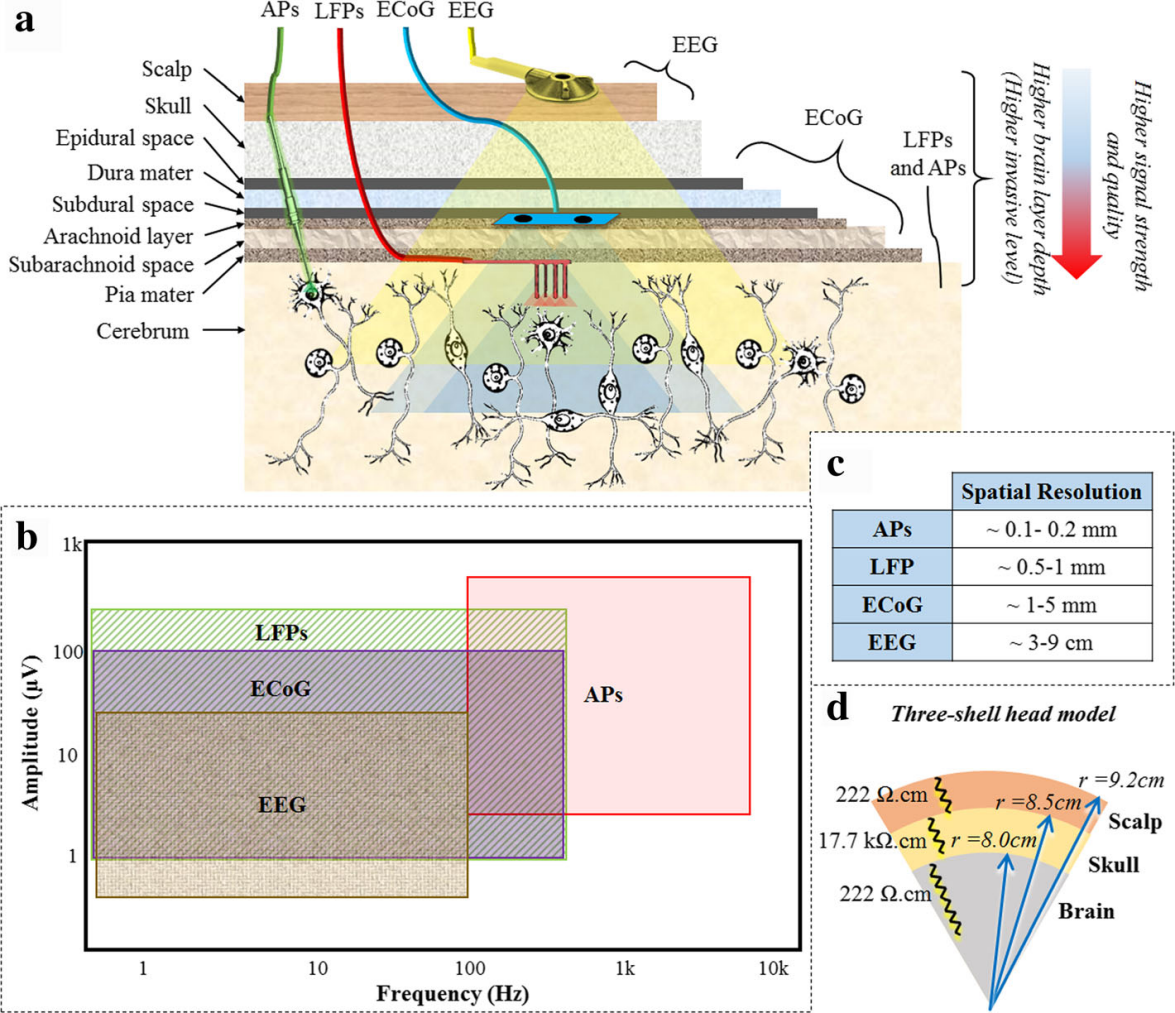
**a** A schematic representing different brain layers and measurable electrophysiological signals. Recording from higher depths results in potentials with higher strength and quality. The higher the distance of electrode from the potential source means a larger impedance. Therefore, proportional to the distance, the potentials are attenuated and high-frequency components are rejected due to the low-pass filtering behavior of the brain layers [159, 160]. In addition, recording from an electrode with smaller contact area enables measuring potentials from fewer neurons [161]. **b** Amplitude vs frequency characteristics of the human brain potentials of interest. **c** The spatial resolution of electrophysiological signals. **d** Three-shell head model. Different layers of the head, particularly the skull with a large resistivity, induce a distorting effect on the potentials that reach the scalp surface

It is crucial for a closed-loop DBS device to take advantage of a feedback signal in the control loop to eliminate the problems associated with open-loop adjustments of stimulation parameters. Open-loop adjustment of stimulation parameters is not an efficient procedure. In the past decade, various physiological signals have been used as a feedback in the closed-loop DBS systems. Action potentials (APs) [2, 22], ECoGs [23, 24], LFPs [3, 12, 25–28], and electroencephalogram (EEGs) [29] are examples of electrophysiological biomarkers considered in the feedback loop of adaptive DBS systems (see Fig. 2 for details on the layer of extraction). Aside from electrophysiological signals, some researchers have focused on creating the feedback loop using other biomarkers such as electromyogram (EMG) [30–33], and biochemical [34] signals. In addition, Hebb et al. proposed the optical and mechanical signals as other possible control signals [9]. However, these signals require further assessments in terms of their practicality. Each biomarker comprises merits and demerits concerning invasiveness, signal content, and resolution, as well as suitability for a disorder type [9] (see Table 1).

**Table 1:** Comparison of several potential biomarkers for the closed-loop DBS

Biomarker	Stability	Invasiveness degree	Capability to merge stimulation/recording electrodes	Patient friendliness	Spatial resolution	Applied diseases
*Neuro-electrophysiological biomarkers*						
EEG potentials	High noise and artifacts sensitivity	Non-invasive	No, needs separate recording and stimulation electrodes	No damage to the head	Poor, ~3–9 cm [38]	PD [29]
ECoG potentials	Moderate noise and artifacts sensitivity	Least invasive	No, needs separate recording and stimulation electrodes	Minor damages to the skull and Dura matter	Moderate, ~0.5 cm [38]	PD and Epilepsy [23]
LFP potentials	Long-term stability [48]	Invasive	Yes, same recording and stimulation electrode [84]	Some neuronal and vasculature damages	High, ~1 mm [38]	PD [3, 12], Epilepsy [86]
AP potentials	Need recalibration for good stability [44], less practical for long-term sue	Most invasive	No, needs separate recording and stimulation electrodes	Extra neuronal and vasculature damages	Very High, ~0.2 mm [38]	PD [2]
*Other biomarkers*						
EMG potentials	High noise and artifacts sensitivity	Non-invasive [31, 32]	No, needs separate recording and stimulation electrodes	No damage to the head	Poor [150]	Movement disorders [30, 33]
Biochemical potentials	Require short time for stabilization of carbon-fiber micro-electrode during recording [45]	Invasive	No, needs separate recording and stimulation electrodes [151]	Some neuronal and vasculature damages	High [152]	Essential tremor [153], and depression [154]

The cortical EEG in PD patients have shown relevance to abnormal basal ganglia circuit functions [29]. In addition, high thalamocortical theta coherence has been verified in PD patients [35]. These features indicate that the cortical EEG signal could be used as a relevant biomarker to the PD symptoms, especially because of its non-invasive nature. However, it suffers from low amplitude (maximum 10–20 μv [36]), low spatial resolution (~3–9 cm [37, 38]), high noise and artifacts sensitivity (e.g. to eye movements [39]), as well as lack of high-frequency components (typically between 0.5–40 Hz [36]). These limitations mainly arise from longer distance of the sensing electrode from the neuronal potential sources. In addition to the stated limitations, the patient’s freedom of movement is restricted through the non-implantable electrodes. Therefore, it causes discomfort to the patient due to the attachment of electrodes to the head.

ECoG as another biomarker has been used by Afshar et al. study [23]. They proposed an ECoG-based brain-machine interface system and presented a correlation of ECoG with the disease state. In comparison with EEG, ECoG has higher signal quality in both amplitude and frequency ranges (maximum 50–100 μv with frequencies between 0.5–500 Hz [36]). This feature has made ECoG a signal with higher popularity in neuroscience research, especially for brain-computer interface (BCI) applications. However, selecting ECoG as a biomarker requires placement of subdural electrodes in the brain epidural or subdural spaces, which are still away from the neuronal stimulation site. Thus, the concerns on the spatial resolution of ECoG signals may still exist. For instance, the spatial resolution deteriorates significantly from 1.25 mm to 1.4 mm by placing the electrode on the epidural instead of subdural space [40, 41]. Moreover, the difference in the recording and stimulation electrodes may yield additional costs and longer implantation and anesthesia duration that may impose further brain injuries.

In contrast, APs, because of their high spatial resolution (maximum 0.2 mm [38]), can be a good candidate as a biomarker for controlling adaptive DBS devices. Typical APs recorded extracellularly have an amplitude of up to 500 μV (100 mV intracellularly [42]) with frequencies between 100 Hz - 7 kHz [43]. However, continuous recording of APs can cause death of neurons (if intracellular). Moreover, there is a need for recalibration processes to keep the stability of feedback signal [44]. Thus, these limitations prevent the use of APs as a biomarker for long-term stimulation. Furthermore, other biomarkers such as biochemical potentials need stabilization of the carbon fiber microelectrode during recording [45]. Furthermore, applying other biomarkers such as EMG seem to have similar limitations as EEG. Moreover, EMG is linked to only a limited number of diseases including PD and essential tremor [30–33].

On the other hand, LFPs are the most used feedback signal in closed-loop DBS [3, 12, 26]. LFPs, also known as intracranial EEG [46], are potentials generated in the extracellular space by propagation of APs through axons. These local potentials reflect neuronal processes occurring within a local region around electrode in the neuronal extracellular space [47]. Priori et al. review [12] demonstrated the suitability of LFPs as the feedback signal in closed-loop DBS devices for PD patients. One key advantage is that LFPs can be directly recorded from the stimulation electrodes. Another advantage of LFPs is the long-term stability achieved at the electrode-tissue interface [48]. LFPs generally have amplitudes of up to 200 μV with energies below 500 Hz [49]. Compared to APs, LFPs have a reasonable spatial resolution, typically around 1 mm [38]. As discussed in a review by Deeb et al. [50], thus far several neurological disorders have been investigated with closed-loop DBS systems controlled by LFPs. While PD and epilepsy have been mainly focused on, other disorders including Tourette syndrome, major depression, and tremor have been also tried recently.

Generally, the selection of a proper biomarker depends on several factors. It is usually chosen with respect to the disease type and the degree of relevance to the disease symptoms. Apart from being linked to a symptom, it is vital for a biomarker to be recorded with a high signal to noise ratio, and most importantly be stable and unaffected by external artifacts such as movement, talking, and thinking [11].

## Control of feedback and stimulation parameters

**Fig. 3:**
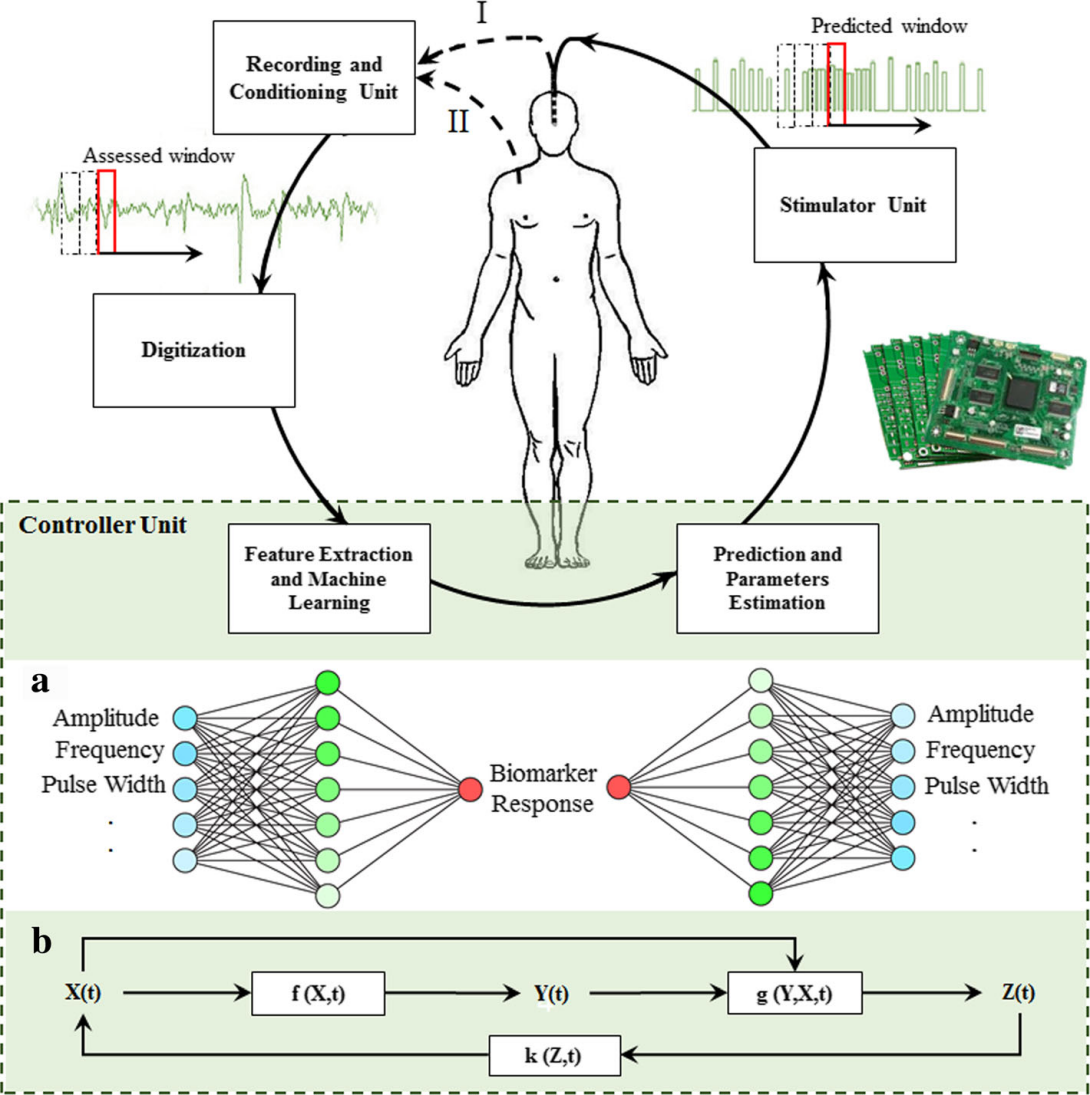
The process of real-time closed-loop DBS programming. The recording unit records the biomarker signal via an inserted electrode inside (I) or outside (II) of the brain based on the biomarker type. After signal conditioning (amplification and filtration), the biomarker signal is digitized and then sent to the controller unit. Then, through a computational model (A), the biomarker signal is evaluated from different aspects (e.g. amplitude, frequency, and pulse-width, etc.) to define the response signal, which is then employed to predict optimized stimulation parameters. The bottom model (B) has been adopted from [105] and then modified. It represents the structure of controller where *X (t)*, *Y (t)*, and *Z (t)* are the input vector, neural circuit states, and biomarker response, respectively. The mapping functions from input to the neural state and from neural state to the biomarker response are demonstrated by *f (X,t)* and *g (X,Y,t)*, respectively. The *k (Z,t)* is the controller that evaluates the biomarker response and updates stimulation parameters. The estimated parameters are adjusted in the stimulation unit to create the stimulation pulses for applying to the stimulation electrodes. In this real-time process, a very short time-window of the recorded signal is used for prediction of the stimulation parameters. The time-window of biomarker signal is pushed forward continuously and simultaneous computations are done to predict and update the next stimulation-window

Automatic and non-subjective optimization of closed-loop stimulation parameters can enhance the patients’ therapeutic benefits while minimizing the side effects. Although the neurologists reprogram the open-loop DBS to enhance the therapeutic results, the procedure is not optimal and would not deliver the best therapeutic effects [2]. Simultaneous and automatic control of stimulation parameters could improve the effectiveness of therapy. A schematic of the closed-loop DBS programming process is illustrated in Fig. 3.

Several neurological diseases are caused by synchronized populations of oscillatory neurons. In some patients, open-loop DBS may not yield desynchronization or its therapeutic effect may decline over time [51]. In particular, the brain reaction to a constant set of stimulation parameters may change gradually over time due to the external (environmental) or internal (disease progress, and behavioral) factors [52, 53]. This procedure is called neural plasticity which is the ability of the nervous system to adopt a new functional or structural state in response to extrinsic and intrinsic factors [54].

During the past decades, several model-based closed-loop stimulation methods have been developed in order to restore desynchronized dynamics in networks of oscillatory neurons. These methods include single-site linear [55, 56], multi-site linear [57, 58], and non-linear [59–62] delayed feedback stimulation techniques, which intrinsically operate in a closed-loop or demand-controlled manners. Another model-based method for desynchronization of oscillatory neurons was proposed by Tukhlina et al. [63] which is an implementation of a phase shifter. In this method, suppression of neural synchrony is realized by organizing an interaction between the ensemble and a passive oscillator [63]. In recent published works [64, 65], Popovych et al. combined the closed-loop DBS approach with desynchronizing stimulation protocols. They extended linear and non-linear delayed feedback stimulation methods to a pulsatile closed-loop DBS, and showed effective and robust desynchronization of STN-GPe model neurons. In addition, they showed that the presence of an interphase gap between the recharging phases of the charge-balanced biphasic pulses can significantly improve the stimulation-induced desynchronization in a closed-loop DBS. Here, the amplitude of the stimulation pulses was adjusted by the LFP feedback signal based on linear or nonlinear delayed feedback rules.

In addition to the above presented works, there exist many other interesting reports on development of an optimized controller for programming of stimulation parameters. Santaniello et al. [66] developed a model-based controller through simulation of intrinsically active neurons in the Vim thalamus and getting LFPs from the neurons. This controller works based on a recursive autoregressive model and adjusts the stimulation amplitude automatically based on the feedback signal recorded from the stimulation electrodes. Pyragas et al. [67] presented a demand-controlled method for suppression of neural synchrony using a proportional-integral-derivative (PID) feedback, which enables robust restoration of desynchronized states. Similarly, Gorzelic et al. [68] and Dunn et al. [69] suggested model-based algorithms that operate based on PID feedback. Their suggested algorithms enable optimization of stimulation parameters in the closed-loop DBS. In addition, an adaptive feedback input-output linearization algorithm for closed-loop control of PD has been proposed by Su et al. [70]. This algorithm adjusts the input signal based on the estimation of parameters from the feedback signal. Rhew et al. [25] designed a log-based adaptive DBS device that utilizes low-pass-filtered LFPs in a proportional-integral (PI) controller to define the optimum stimulation amplitudes. This system works based on biomarker energy and benefits from a robust feedback controller because of removing high-frequency artifacts from the controller input. Another interesting controller has been designed that operates based on variations in the neurochemical release [34]. The integration of recording and stimulation modules is facilitated via fast-scan cyclic voltammetry (FSCV). This system records artifact-free evoked neurochemical alterations to wirelessly control the stimulation parameters [34]. Another neurochemical controller is proposed by Grahn et al. [71] in which evoked dopamine changes are recorded to adapt the stimulation parameters in a rodent model.

Some of these closed-loop controllers, however, only control one of the pulse parameters (usually amplitude) or just implement a simple ON-OFF control of stimulations. The on-demand systems trigger the DBS switch to act only when the stimulation is needed. It is a simplified form of closed-loop DBS capable of preventing brain over-stimulation. The idea is to set a threshold and check the biomarker continually, finding when it crosses the threshold to turn on or off the stimulations. This type of strategy to control the stimulation pulses in a closed-loop DBS is based on an “*amplitude-responsive*” strategy [72]. Numerous studies focus on the increment of beta frequency power (13–35 Hz) in PD and tremor [73–75]. Little et al. [3, 76, 77] designed an on-demand adaptive DBS that digitally filters the biomarker around the beta frequency (22 Hz), generating a smoothed beta amplitude. Then, they utilize it as an input to the controller that regularly checks the beta oscillatory amplitude with respect to a personalized threshold (different in each patient). Instead of providing an on–off strategy, Rosa et al. [78] presented an algorithm that continuously and linearly modifies the stimulation parameters each second according to the changes in the patient’s LFP beta power (13–17 Hz).

One of the concerns about the use of the beta frequency band as a feedback biomarker is that it might be affected and suppressed by movement [79]. This issue is not an important problem in the experimental studies when the patient is motionless. However, in a beta-based closed-loop DBS device implanted in the patient’s brain, the patient’s movements can supress the beta amplitude which may degrade the closed-loop performance. Another concern relates to the accuracy of the promising outcomes of beta-based studies due to the fact that they only test the patients in a temporary time period (after electrode implantation and before surgery to implant the pulse generator, when the leads are accessible). This period, known as post-operative “stun effect”, is believed to causes unrealistic outcomes as a result of temporary alleviation of PD symptoms even without stimulation [80, 81]. However, a recent published paper [82] presents a proof of principle to the chronic application of adaptive DBS, and confirms the accuracy of the closed-loop DBS in the temporary period. Piña-Fuentes et al. [82] presented the first case of closed-loop DBS in a PD patient with chronic STN-DBS treatment, and concluded that closed-loop DBS can be applied in the chronically implanted DBS phase, and is at least as effective as open-loop DBS when objectively assessed [82].

Beyond the “amplitude-responsive” strategy for controlling the stimulation pulses in a closed-loop DBS, there is another strategy termed “phase-responsive”. In a phase responsive closed loop DBS, the stimulations are directed by the phase (timing) of the biomarker signal [72]. The phase-responsive closed-loop DBS is currently being developed for treatment of tremor, aiming to selectively stimulate at the phases that attenuate the tremor amplitude through an accelerometer attached to the tremulous hand [72, 83]. The results of this control strategy show a significant tremor relief in essential tremor patients [83]. Thus far, the phase-responsive closed-loop DBS has not been applied for longer than 30 s [72].

## Technological advancements towards closed-loop DBS devices

In this section, the existing closed-loop DBS systems both from hardware (circuit design) and software (control algorithm and programming) perspectives are reviewed.

### Stimulation artifact suppression

The stimulation current pulses can cause interference with reading of biomarkers of interest. This interference makes it difficult to accurately record and process biomarkers because of the large difference in the amplitude of the stimulation pulses and the measured signal. For instance, the magnitude of LFPs is roughly five to six fold (100–120 dB) smaller than that of the stimulation pulses [84]. This difference yields a combination of strong stimulation artifact (in volts range) with a weak neural signal (in μV range), which saturates the amplifiers used in the recorder.

To alleviate this problem, Rossi et al. [84] designed “FilterDBS” which is an artifact-free recording system for acquisition of LFPs from the DBS lead positioned in the STN. The 130 Hz stimulation artifact and the higher harmonics were separated in the frequency domain using a bandpass filter (2–40 Hz). This system benefits from an overall gain of 100 dB with 130 dB common mode rejection ratio (CMRR). However, the device requires a ± 15 V supply to operate. Post-filtering is another method to remove the stimulation artifact [85], in which the template of the stimulation signal is subtracted from the recorded signal to produce an artifact-free biomarker. However, this method degrades the signal quality which is not desirable. Moreover, it may not operate correctly in the closed-loop DBS where the stimulation rate fluctuates.

Stanslaski et al. [86] designed an implantable, chronic, adaptive DBS device that benefits from an LFP/ECoG sensor. This device was validated successfully in an ovine model of epilepsy. The hippocampus seizure activity was detected and measured during and after stimulation. The separation of the biomarker from the stimulation artifact was conducted through a support vector machine classification algorithm by processing of the spectral fluctuations. The suggested device fits in a 39cm^3^ space, employing front-end filtering that guarantees the op-amp input to be within its normal operation area [86]. In another work, Zbrzeski et al. [87] introduced an integrated neural amplifier to reduce artifacts in the recording of LFPs and spikes. The proposed neural amplifier occupies a space of 0.15 mm^2^ and consumes 6.73 μW of power. Test-bench validation of the amplifier showed a mid-band gain of 20 dB with a low input-referred noise of 4 μV_rms_. However, despite the advances in the simultaneous recording and stimulation, the issue of stimulation artifact in the recorded signal has not been fully addressed yet in the existing DBS systems.

### Stimulation challenges

#### A. Stimulation pattern

**Fig. 4:**
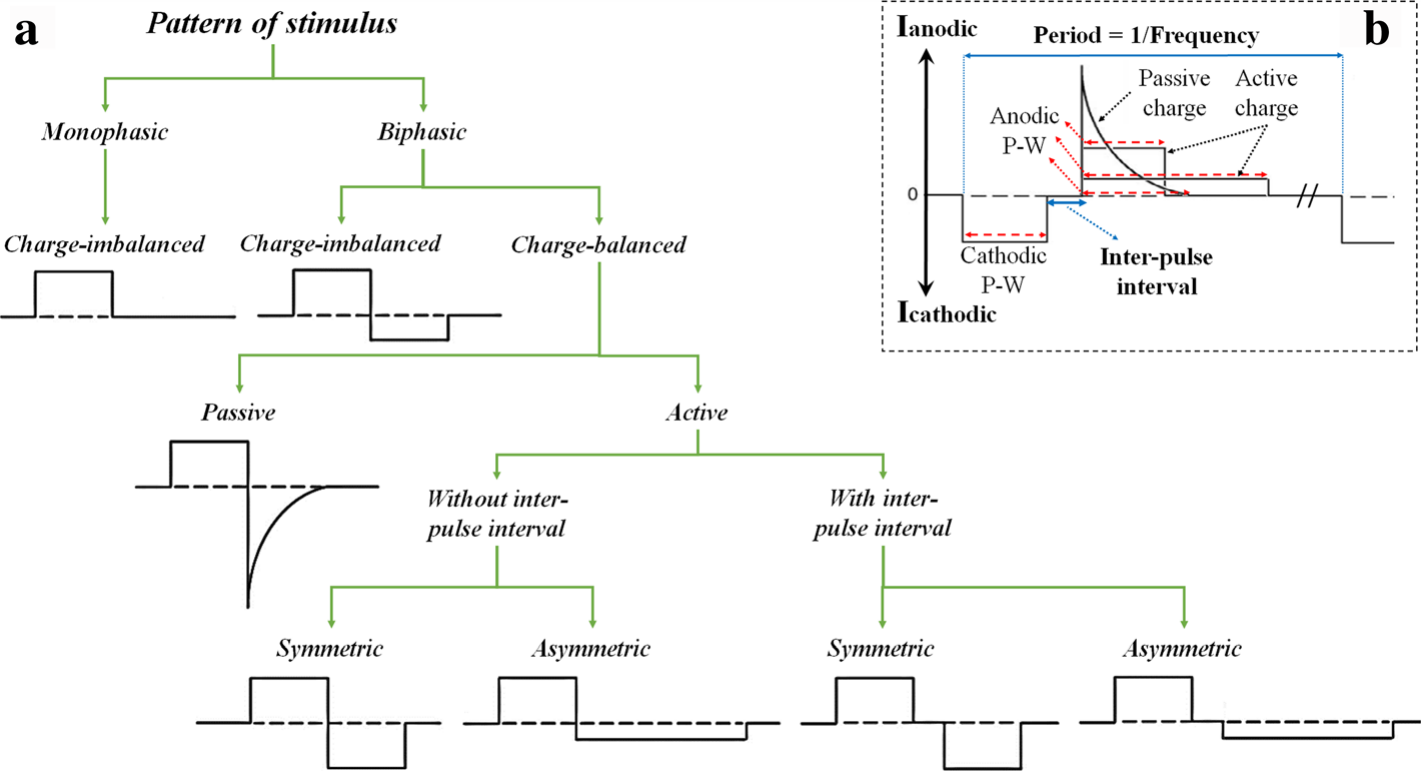
Categorization of different stimulation patterns. For additional details regarding the pros and cons of each waveform refer to [89, 162]

The pattern of stimulation is a major concern for both open-loop and closed-loop DBS devices. Figure 4 shows the differences between monophasic (a fully positive (cathodic) or a fully negative (anodic) pulse) versus biphasic (a pulse having both anodic and cathodic sides) stimulations. Whilst monophasic pulses are charge-imbalanced, biphasic pulses can be charge-imbalanced or charge-balanced. Being imbalanced in terms of charge yields formation of undesirable chemical reactions at the electrode contact surface over time [88]. Therefore, monophasic and charge-imbalanced biphasic pulses can be dangerous for the brain tissue due to their non-zero net charge that can create a lesion. Charge-balanced pulses can be obtained through passive or active charge balancing schemes. In the active methods, the second pulse is truly built using a second current source or through a further complex circuitry with one current source. However, in the passive charge-balancing scheme, a passive components or shortening of the electrodes to the ground is used. Although charge-balanced biphasic pulses may prevent net charge injection into the stimulated tissue [88], the anodic phase neutralizes the anticipated stimulation effect of the cathodic phase [89]. Typically, an inter-pulse interval is applied to the biphasic pulses in order to avoid cells hyperpolarization and minimize the suppressing and altering effects of the anodic phase [89]. It is worth stating that most of the available market-based open-loop and closed-loop DBS systems use a passive charge-balancing scheme.

Ewing et al. proposed a DBS device named SaBer DBS [90]. It delivers biphasic, passive charge-balanced pulses to two independent channels. Immediately after delivering the stimulation pulses, the electrodes are grounded (capacitive coupling) to achieve the zero-net charge. Although it is programmable in frequency, pulse-width, and current amplitude, the user defines the stimulation parameters; thus, the system is not closed-loop. Moreover, lack of inter-pulse interval between the cathodic and anodic pulses may alter neuronal activities. Sooksood et al. [89] proposed a novel active approach for charge balancing via long-term offset regulation. Using this method, the voltage of the electrode is checked after each stimulation pulse to fall within a predefined range. If not, the mismatch is compensated via an adjusted offset current. The power-efficient DBS system designed by Lee et al. [91] uses the offset regulation method. They designed a power-efficient DBS system that employs closed-loop active charge balancing scheme (by utilizing a small balancing current pulse) to retain the charge within a safe window (50 mV). It is also capable of estimating the residual time remained for balancing.

Apart from the pattern shape, a temporally irregular (i.e. variable inter-burst interval) stimulation pattern in the closed-loop DBS has caused concerns regarding the effectiveness of stimulation. On one hand, it has been observed that irregular stimulation patterns can reduce the beneficial therapeutic effects [92, 93]. On the other hand, the closed-loop DBS appears to be irregular because of the adaptive variations. Rosin et al. [2] applied both closed-loop stimulation and random stimulation (unrelated to the ongoing activity) patterns in two PD monkeys. They observed that in spite of the irregular pattern of the closed-loop DBS, it is a successful therapeutic method. It was explained that the adaptive (symptom-related) nature of the closed-loop DBS induces the therapeutic effects regardless of its pattern’s irregularity.

#### B. Stimulation type

Delivering method of stimulation pulses, whether to dispatch constant-current-controlled (CC) or constant-voltage-controlled (CV) stimulation, is a matter of debate. Most of the available commercial DBS devices offer the CV stimulators due to their higher level of power efficiency [14]. In the CC stimulation, the dropout voltage across the current source wastes the power and reduces the device battery life. On the other hand, the current and the volume of the tissue activated in CV devices may alter because of the impedance variations in the brain tissue and the electrode-tissue interface [94]. Whereas the CC stimulators provide a more precise control independent of the impedance variations. Impedance fluctuations have been observed during the first 3 months following DBS surgery [95, 96] as well as post-implantation [26, 97, 98]. Satzer et al. [99] highlight a mean 73 Ω/year reduction in impedance in most electrode contacts; thus, utilizing CC devices is more preferred. However, in terms of effectiveness, there is no proven evidence to give CC preference over CV stimulation [100]. Although better clinical outcomes were reported via CC stimulation in patients suffering from dystonia in the Lettieri et al. experiment [94], the study had some limitations as discussed in ref. [101].

In order to improve the power efficiency problem in CC stimulators, Azin et al. [102] proposed a closed-loop DBS device which controls the stimulator supply voltage adaptively through an inductive link. It is a 4-channel intra-cortical micro-stimulation integrated circuit (IC) (10.9 mm^2^ system on chip (SoC)) that converts recorded extracellular neural spikes to electrical stimulations for real-time delivering from one brain region to another [102]. It comprises a voltage readout channel to close the feedback loop. Moreover, it includes a direct current (DC) to DC converter that converts a single 1.5 V battery to 5.05 V to supply the micro-stimulating back-end circuit. Similarly, Hyung-Min et al. [91] designed a 4-channel wireless CC stimulator with a closed-loop supply control that utilizes a voltage readout channel. The voltage readout channel automatically controls the stimulation voltage and improves the efficiency (30% higher than a fixed supply voltage). This prototype has been verified in vitro, occupies 2.25 mm^2^ with 5 V peak alternating current (AC) input (rectified to a DC voltage between 2.5 V to 4.6 V) at 2 MHz.

### Portable closed-loop DBS

Another issue is the size and weight of the closed-loop DBS device. Compared to the open-loop DBS devices, closed-loop systems have additional recording and programming circuits which increase the size and weight of the final design. Because the initial experiments are usually carried out in small laboratory animals, it is necessary that the device be miniature and lightweight, allowing stimulation during the animal life cycle (eating, playing, walking, sleeping, etc.). The issue is even more important when the device is expected to be used for long-term experiments. Providing a balance of functionality, portability, the number of input and output channels, and power requirements in the closed-loop DBS devices demand a comprehensive research.

Jongwoo et al. [103] developed a 64-channel programmable IC to be used as closed-loop DBS device for treatment of neurological disorders. This device employs eight low-noise front-end pre-amplifiers, single 200 kS/s 8-bit logarithmic pipeline analog to digital converter (ADC), and digital filters. It has been implemented in 0.18 μm complementary metal–oxide–semiconductor (CMOS) technology and benefits from 2.7 mm^2^ occupied space. Both LFPs and spikes are sensed and then separated through on-chip digital filters. The proposed device consumes 89 μW in a normal mode and 271 μW in a configuration mode from 1.8 V supply. In addition, Pinnell et al. [104] developed a miniature wireless closed-loop CC DBS with a total weight of 8.5 g to be used with laboratory animal. This system operates for more than 8 h and transmits LFPs to 3–5 m distances. Another recent effort towards the portability is the work done by Arlotti et al. [16]. They have developed an external portable closed-loop DBS for clinical experiments in freely-moving PD patients. This device analyses LFPs recorded from a single differential artifact-free channel. The external part weights 150 g occupying 12 × 7 × 2.5 cm^3^ in space. It can be worn externally by humans to investigate closed-loop DBS effectiveness. However, it is a large and heavy device for small laboratory animals.

### Battery-less closed-loop DBS

Another hurdle in designing a closed-loop DBS is the possibility of continuous biomarker recording and real-time processing with least energy consumption. Some researchers have developed DBS devices that consume low power [105–107]. However, these devices have limitations with lack of re-chargeability and limited memory space. These weaknesses can impose potential surgery risks and extra costs on patients [108].

The invention of energy harvesting technology has increased the battery lifetime of DBS devices, reducing the frequency of surgical replacement procedures, and subsequently minimizing the extra costs. Harvesting the power from human or environmental sources (kinetic, electromagnetic, thermal and infrared radiant energies) have been discussed in a review by Hannan et al. [109]. The review suggests the inductive coupling link as a more suitable method of powering the battery-less implantable biomedical devices. Harrison et al. [42] designed a neural recording system in which power and command are transferred wirelessly (at 6.5 kb/s) via a 2.64 MHz inductive link. In other studies, Hosain et al. [110–112] proposed a four-layer circular planar inverted-F antenna to harvest power for adaptive DBS implants and examined the feasibility of wireless transmission of power, control, and command signals. They then proposed RF rectifiers for electromagnetic power harvesting in a DBS implant [113]. Besides these, a system-on-chip wireless closed-loop DBS was developed by Hyo-Gyuem et al. [25, 114] which is capable of two-way wireless telemetry through RF energy harvesting. Lin et al. [115] proposed another battery-less, implantable neuro-electronic interface capable of two-way wireless telemetry. It receives power through a single coil and transmits data via the same coil. The device has been fabricated with the standard 0.18 μm CMOS technology with a chip area of 7.74 mm.

### Patient-friendly closed-loop DBS monitoring

Another demand is providing a more patient-friendly closed-loop DBS device by making commands through wireless data communications for home care facilities. Early detection of DBS side effects could result in immediate action of neurologists in modifying stimulation parameters. This cannot be achieved unless through a long-term and ongoing monitoring of the disease state. Even in the closed-loop DBS where programming is done automatically, the need for monitoring the stimulator related side-effects exists [116]. Apart from the stimulator’s side effects, the battery capacity needs to be monitored after implantation to avoid hazards of battery depletion [116].

Upon development of information technology, telemedicine is progressively applied for various medical applications. However, only a few programs have focused on connecting DBS devices to the hospital networks. A web-based platform (WebBioBank) for neurophysiological DBS data collection has been developed for integration of PD clinical data [117, 118]. This system creates a reference guideline for home care and monitoring of DBS patients. It is capable of connecting to patient’s smartphone and safely share the information to improve the care quality for DBS patients. However, such an integrated system is in the early stages of development and requires further research to become available not only to PD patients but also to other treatable disorders via DBS. In another study, Chen et al. [119] first identified the DBS telemedicine requirements through a questionnaire responded by 22 patients and 9 neurosurgeons. The results indicated that providing of an urgent remote adjustment was needed. Moreover, the preferred communication method was the video telehealth interaction. According to their findings, adding an interaction mode to the DBS systems was proposed. Then, they successfully tested the functionality of the idea on three patients.

## Commercial closed-loop DBS devices

DBS devices have been in commercial use since 1997 when the food and drug administration (FDA) organization approved the utilization of neurostimulator devices for tremor treatment [120]. Since then, they have been also approved for other disorders such as PD in 2002 [121], dystonia in 2003 [122] and obsessive-compulsive disorder (OCD) in 2009 [123]. The Activa system by Medtronic (Minneapolis, MN, USA) is a widespread open-loop DBS device. However, other companies such as Boston Scientific and St. Jude Medical also manufactured comparable products.

The study of the relevant literature shows that there is currently only one commercially available brain stimulation system that provides closed-loop DBS (The closed-loop RNS system by NeuroPace for closed-loop cortical stimulation). The closed-loop RNS system can stimulate depth and/or cortical tissues of the brain using two different types of leads. It is a responsive battery powered closed-loop DBS capable of continuous sensing of ECoG potentials. The RNS system has received FDA approval to be used as an alternative option for treatment of drug-resistant epilepsy patients [124]. Upon recognition of a predefined pattern relating to seizure in the recorded signal, the stimulator is activated to remove the pattern. This device stores segments of ECoG potentials. In addition, it can be programmed to store information about the detected electrographic events (e.g. the date and time of seizure occurrence).

Another commercial closed-loop DBS system is the Activa PC + S which is an implantable sensing-stimulating DBS device manufactured by Medtronic. This device has been designed for investigational purposes. The PC + S system can provide concurrent LFP sensing and stimulation. However, it does not use the sensed information for adjusting the stimulation pulses [125], unless the user compiles a program into the device and closes the feedback loop. If Medtronic provides an upgrade for the PC + S system with all the necessary programs for closing the feedback loop, then, the Activa PC + S system can function as a complete closed-loop DBS device.

## Discussions

### Weakness of open-loop DBS devices

Once a conventional DBS system is implanted, the stimulation parameters need to be defined. The procedure in which the optimum stimulation parameters are defined per each subject is a trial-and-error-based programming task that is carried out by clinicians. Using a set of guidelines, the stimulation parameters are changed based on the observable behavioral responses to the previous stimulation parameters [126]. Unfortunately, this procedure is a subject-dependent and time-consuming task that may frustrates both the clinician and patient.

Although the neurologists reprogram the open-loop DBS to enhance the therapeutic results, this reprogramming is not an optimal method. Even by achieving an optimum set of parameters in one effort, there is no guarantee that the symptom relief response will last long due to the brain neural plasticity. Neural plasticity usually occurs after prolonged DBS resulting in reduced efficacy over time. For instance, Ricchi et al. [127] observed that the gait improvement obtained by switching the stimulation frequency from 130 Hz to 80 Hz was not achievable at follow-up sessions. Occasionally, it is feasible to recapture the efficacy of open-loop DBS using a coordinated reset (CR) stimulation [128, 129], which is a short-duration stimulation interval for rewiring of neuronal networks [130]. Many computational studies reported optimization of CR stimulation so that long lasting therapeutic effects can be achieved [64, 65, 131]. The results were obtained in rat hippocampal slices [132], MPTP-treated monkeys [133, 134], human PD patients [135] as well as in tinnitus patients [136, 137]. The outcomes confirm the robustness of CR open-loop stimulation for the treatment of neurological disorders characterized by abnormal neuronal synchronization [131]. Note, the application of CR stimulation is not limited to open-loop DBS, and can also be used with closed-loop DBS in a demand-controlled manner [128, 129, 138] to fight against induced neural plasticity over time.

Furthermore, the open-loop programming procedure may not be practicable for all kind of neural disorders. For instance, the therapeutic response to the programming of stimulation parameters could take several weeks to finalize for depression or dystonia [71, 139], while being instantly evident for epilepsy, tremor, and PD [71]. Taking a longer time for appearance of the therapeutic response makes it hard for neurologists to decide whether the adjusted parameters are superior to the previous settings or not. Therefore, the open-loop programming could be inefficient for certain neurological disorders.

Another weakness of open-loop DBS devices is that its frequent programming sessions incur additional costs to the patients. Patients normally don’t like the numerous clinic visits required for adjustments of parameters to optimize their symptom relief response. In addition, reprogramming of open-loop DBS devices needs regular involvement of clinical experts. Therefore, this could be an extra burden on clinics to employ and instruct clinical staffs to train them skills in open-loop DBS programming [140].

In addition, open-loop DBS devices are faced with the issues associated with battery life due to their continuous and non-stop stimulation pulses. One issue is that battery replacement requires a surgery which imposes potential risks (e.g. surgery risks, anesthesia risks) to the patient [3].

In summary, open-loop DBS therapy has several weaknesses including: (1) subject dependency, (2) time-consuming programming, (3) lack of dynamical adjustment of stimulation parameters, (4) frequent visits to clinic for programming, (5) extra costs in visits to clinic, (6) inefficiency for some neurological disorders (e.g. disorders with a lag response to the stimulation), (7) regular involvement of programming experts, and (8) short battery life in battery-operated devices. Hence, the open-loop programming of DBS devices is not optimal method and might not result in the best therapeutic effects.

### Comparison of closed-loop DBS devices

A comparison of the existing closed-loop DBS devices is presented in Table 2. It provides an evaluation of the existing device from different technical standpoints. Although these devices have common functional features, they incorporate different monitoring and stimulation designs, control methods, and internal features. Some of these devices close the feedback loop by turning the stimulation on and off known as on-demand stimulation [31, 32, 104, 141]. The stimulation is delivered only when the abnormal phase of biomarker is detected. While other devices take a step forward and update at least one stimulation parameter based on the biomarker state, usually pulse amplitude [86, 102, 114]. The existing closed-loop DBS devices (as compared in Table 2) employ only one biomarker in the control unit to decide on the adjustment of stimulation parameters. While some of the devices can be used to record more than one biomarker (e.g. LFPs and APs), but they cannot detect and process multiple biomarkers concurrently. Therefore, only one biomarker is used as a feedback to control the DBS pulse generator. Using only one biomarker to close the feedback loop has the disadvantage of being affected by noise and artifacts. Occasionally, due to the device internal or external environmental conditions, the biomarker and the electrodes can be affected by some unexpected noises and artifacts. These unexpected conditions include, but not limited to, magnetic field interactions, induced electric currents, temperature variations, device functional disruptions [142, 143]. If the device is affected by one of the stated conditions, the biomarker will be affected and a wrong decision will be made by the controller. Consequently, the DBS unit will adjust the stimulation parameters unrelated to the variations in the patient clinical state. Being unrelated to the disease may lead to either opposing pathological effects or insufficient treatment. Moreover, the neurons may get damaged because of the incorrect adjustment of the stimulation parameters [144]. One solution is to provide the controller with multiple feedback signals [145]. Whenever a fault occurs in one biomarker sensing path, there will be another path to steer the closed-loop operation. Therefore, this could increase the robustness of the closed-loop DBS device and decrease the chance of inaccurate adjustment of stimulation parameters.

**Table 2:** A comparison of the features of the existing closed-loop DBS devices

*Reference*	[86]	[103]	[102]	[104]	[155]	[114]	[34, 156]	[157, 158]
Recorder and Controller								
Biomarker	LFPs/ECoG	LFPs and APs	LFPs and APs	LFPs	LFPs	LFPs	FSCV	EEG/ECoG
Disease type	Epilepsy	PD	PD	No special disorder, for behavioral analysis on rodents	PD	A generic design (no in-vivo validation)	PD	Epilepsy
Sensing channels	1	8	4	4	1	4	2	64
Controllable parameters and programming method	Amplitude, by support vector machine algorithm	Amplitude, P-W, and frequency	Pulse amplitude using spikes discrimination algorithm	On-demand, DBS ON and DBS OFF commands by PSD calculations	On demand, ON-OFF operation, Evodopa-induced modulations of the LFP beta was used to control the stimulation parameters	Amplitude by LFP energy calculation using a programmable PI-controller	Triangle wave scanning between −0.4 and 1.5 V at a scan rate of either 400 or 1000 V/s, to monitor changes in dopamine	Based on a high-efficacy signal processing algorithm, abnormal phase synchrony triggers the biphasic stimulations
C-SS	Yes	Yes	Yes	Yes	Yes	NS	Yes	NS
Input referred noise	NS	5.29 μV_rms_	3.12 μV_rms_	NS	NS	6.3 μV_rms_	NA	5.1 μV_rms_
Noise efficiency factor	NS	NS	2.68	NS	NS	3.76	NA	4.4
CMRR/PSRR	>80 dB	NS	>56 dB	NS	>100 dB	NS	NA	75 dB
Gain	-	44 dB	~ 52–66 dB	520 linear	80 dB	54 dB	NA	54–60 dB
Analog filters	dc-8 to 500 Hz	0.5 Hz – 10 kHz	1.1 Hz – 10 k Hz	1.5–100 Hz	2–40 Hz	0.64 Hz-6 kHz	NA	1 Hz-5 kHz
Power consumption	100 μW per channel	9 μW per channel	26.9 μW per channel	NS	NS	245 μW	NS	10 μW/channel, 1.4 mW P-D
Stimulator								
Stimulation type	CV	CC	CC	CC	CV	CC	CV and CC	CC
Stimulation parameters (amplitude, pulse-width, frequency/period)	A: 0–3.5 V, P-W: 90 μs, F: 120 Hz (Activa PC DBS was used)	A: 99 μA default (max 135 μA), P-W: 5–320 μs in 5 μs steps, F: 31 Hz to 1 kHz (130 Hz default)	A: 0–95 μA (anodic) and 0–32 μA (cathodic), P-W: 0–240 μs (anodic) and 0–720 μs (cathodic)	A: 100 μA, P-W: 10 μS–500 mS (200 μS default), F: 0.1 Hz–5 KHz (130 Hz default)	A: 2.2 V, F: 130 Hz, P-W: 60 μs (max 200 μs)	General channels: A: 0–116 μA, P-W: 1 ms, P: 65 ms. High-current channels: A: 0–4.2 mA, P-W: 1 ms, P: 65 ms.	A: 50 mV-10 V in VRM and 10 μA-10 mA in CRM, P-W: 50 μs-2 ms, F: 30–120 Hz	A: 0.1–1.2 mA, F: 1–200 Hz, P-W: 40–1000 μs.
Stimulation pattern	Biphasic, passive charge-balanced	Biphasic, passive charge-balanced	Can be either active or passive charge-balanced, user selects	Monophasic, charge-imbalanced	Biphasic, asymmetric charge-balanced	Biphasic, symmetric charge-balanced	Can be either monophasic or biphasic, asymmetrically balanced	Biphasic, symmetric charge-balanced
Stimulation channels	8 for bilateral stimulation	64	4	2	1	6 general-purpose and 2 high-current	4	64
ADC	Linear	8-bit log pipeline logarithmic	10-bit SAR ADCs	16-bit	12-bit ADC	logarithmic	10-bit ADC	7.6-bit SAR ADC
Amplitude resolution	NS	NS	6-bit resolution	16-bit	NS	6-bit resolution	NS	8-bit
Sampling rate/frequency	422 Hz	200 kS/s	35.7 kS/s/ch	500 Hz	NS	100 kS/s	100 kS/s	Up to 100 kS/s
Power consumption	NS	7.4 μW per channel	NS	NS	NS	138 μW	NS	1.5 mW P-D
Other general specifications								
Working duration	NS	68 h (923 h) in configuration (stimulation-only operation) mode	NS	6–8 h (recording), 50 h (stimulation)	NS	NS	When fully charged: at least 3 h continuous operation	NS
Total power consumption	NS	89 μW in normal and 271 μW in configuration modes	375 μW	20 mA average current consumption	NS	468 μW	NS	NS
Size	39 cm^3^	19 mm × 27 mm, 0.18 μm CMOS 2.7 mm^2^	10.9 mm^2^ SoC	28 mm × 17 mm × 7 mm	12 cm × 7 cm × 2.5 cm	2 mm × 2 mm, 180 nm CMOS	NS	4 mm × 3 mm, 0.13 μm CMOS
Weight including battery	NS	NS	NS	8.5 g	150 g	NS	NS	NS
Power supply	1.7–2.2 V	1.8 V	1.5 V	3 V	Two 1.5 V type AA batteries	5 V	rechargeable 740-mAh lithium-polymer battery	1.2 V recording and 3.3 V stimulation boards
Other features	Fully Implantable, telemetry ability	-	Converting extracellular spikes to electrical stimulations, data telemetry	wireless system, reliable transmission of data across 3–5 m distances	It is an external portable a DBS device	With wireless telemetry and wireless power management	Wirelessly controlled	Ultra-wideband wireless neural vector analyzer

*NS* Not stated, *NA* Not applicable, *C-SS* Concurrent Sensing and Stimulation, *A* Amplitude, *P-W* Pulse-Width, *F* Frequency, *P* Period, *FSCV* Fast-scan cyclic voltammetry, *VRM* voltage-regulated mode, *CRM* current-regulated mode, *P-D* Power Dissipation, *CC* constant current, *CV* constant voltage, *PD* Parkinson disease

### Issues with closed-loop DBS devices

**Fig. 5:**
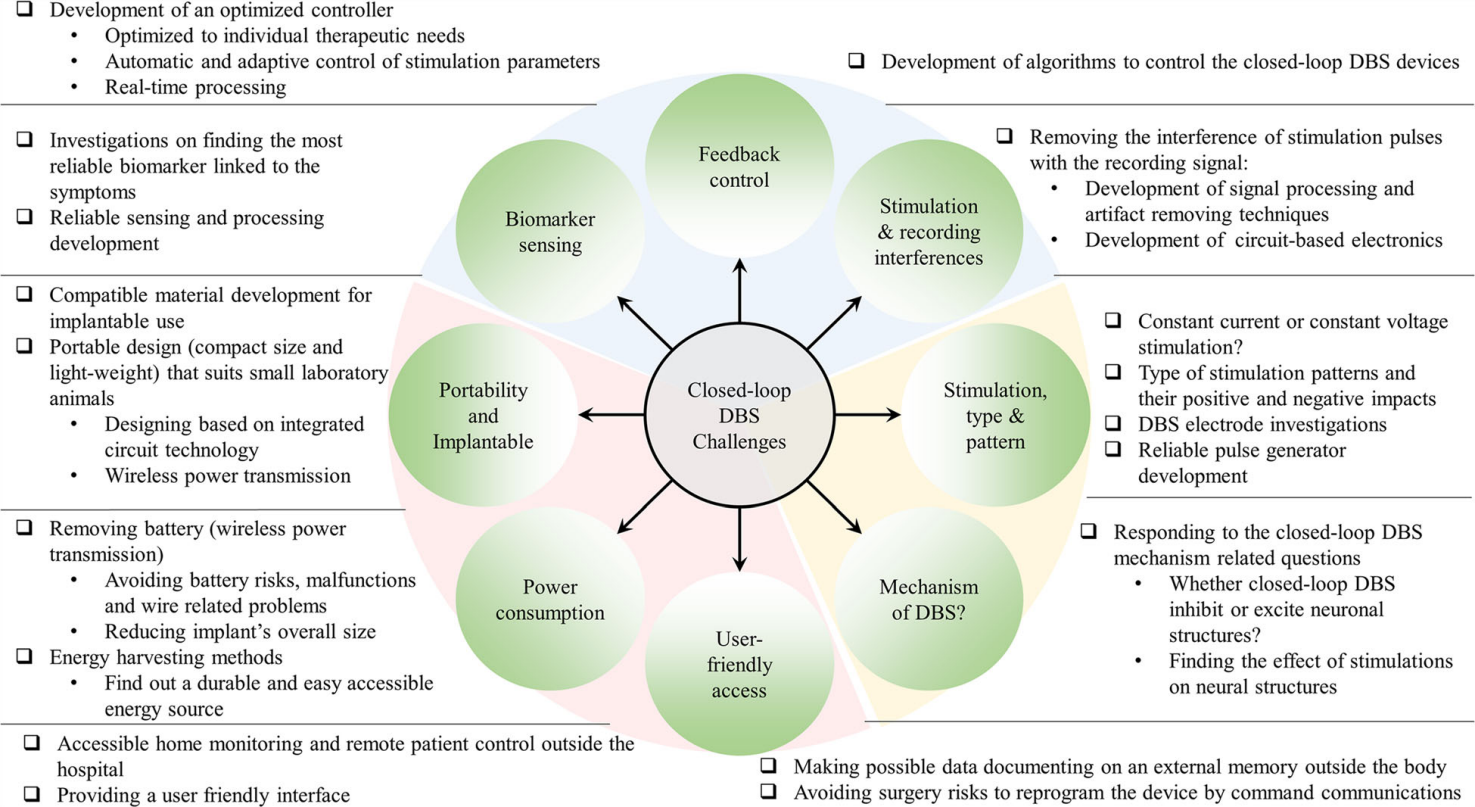
Closed-loop DBS research challenges. These challenges are classifiable in three major parts including monitoring issues (*blue part*), stimulation challenges (*yellow part*), and design expectation concerns (*red part*)

While closed-loop DBS has shown superiority in improvement of clinical outcomes [2], it faces several issues (summarized in Fig. 5). One of the challenges is finding a reliable biomarker linked to the patient’s symptoms. Although several physiological and biochemical biomarkers have been introduced for closing the feedback loop, some of the biomarkers such as LFPs, AP, ECoG, and EEG are affected by the stimulation pulses. Thus, the detection of the biomarker is affected requiring sophisticated artifact removing circuitry to improve the detection of the biomarker. Thus far, several artifact rejection techniques have been developed to tackle the impact of stimulation on biomarker recording [84, 86, 87, 146]; however, the issue has been resolved partially in the current closed-loop DBS devices.

Another challenge is related to the type of stimulation. The clinical difference between CC and CV stimulation needs to be investigated to find out which approach is more advantageous. The pattern of stimulation is another concern due to the fact that a charge-imbalanced pulse would cause damages to the brain tissue. Most of the available market-based open-loop and closed-loop DBS systems use a passive charge-balancing scheme, requiring further investigation and upgrade to an active charge-balancing scheme in order to avoid the weaknesses of the passive charge-balancing as described by Sooksood et al. [89]. On one hand, because of safety issues, the pattern of stimulation pulses needs to be biphasic. On the other hand, biphasic pulses can alter neural activities and lead to suppressing effects on the action potentials. As a consequence, more studies need to explore the clinical impact of charge-balanced and charge-imbalanced stimulation pulses in the closed-loop DBS devices. In addition, the role of stimulation patterns will need to be investigated to be able to answer the remaining questions on the mechanisms and benefits of DBS.

In order to evaluate the effectiveness of the closed-loop DBS devices or examine new applications, the devices need to be validated in-vivo. Due to the small size of most of laboratory animals and also because of the need for long-term experiments, the devices need to have compact size and light-weight, be portable and implantable, and have a tetherless (wireless) configuration to not disturb animal freedom of movement. These are considered as other challenges in designing a closed-loop DBS device. Since closed-loop DBS devices have recording circuitry, the total size and weight of the device may be affected.

In addition, in terms of power consumption, closed-loop DBS devices are expected to consume less power compared with their open-loop counterparts. The reason is that closed-loop devices deliver electrical stimulation based on the state of the brain. Therefore, these devices remain less active over a course of time which results in a reduction of the electrical energy delivered to the brain [4]. However, closed-loop devices have extra recording and processing circuits that could increase the overall power consumption of the device. Therefore, the potential power saving of these devices would depend on the use of low-power recording and analysis circuits.

Although closed-loop DBS devices have a control algorithm that automatically modifies the stimulation parameters according to the patient’s brain state, they may still require regular assessments of the stimulator related side-effects and, if necessary, reprogramming of the control algorithm for minimization of adverse-effects. Developing an optimal control algorithm and/or discovering an optimum biomarker individualized to each patient can take a long time. Therefore, the patients are required to pay extra costs associated with the service. Designing a patient-friendly closed-loop DBS device is another requirement that deserves consideration. This means that providing wireless data and command communications for home care applications is needed to reduce costs and improve care quality.

There are also other concerns associated with the closed-loop DBS therapy, one of them being the effect of pharmacological approaches on the nature and magnitude of measured biomarkers of interest. Simultaneous medication (either oral or injection) with DBS therapy could affect the neuronal activities and change the amplitude of the recorded biomarker. As an example, it has been observed that beta activity is suppressed with levodopa treatment in PD patients [80]. In addition, it has been also demonstrated that DBS could suppress beta activity in off-medication state [147]. However, the patterns of beta oscillations following DBS, differ from those after medication [148]. Simultaneous pharmacological intervention could potentially attempt to change the closed-loop DBS results when applied. Besides the pharmacological intervention, the interaction of progressive severity of the disease may affect the measured biomarkers. Very little work has been done to demonstrate a correlation between a particular biomarker and disease severity over time. In the presence of an opposite correlation, this factor might pose a problem for the use of biomarker in the feedback loop. Therefore, it reinforces the use of individualized biomarkers based on patient’s clinical phenotype or use of combination of biomarkers [48].

Another concern is alleviation of a range of disease symptoms using a single biomarker (or multiple biomarkers). Take PD for instance, each patient may experience different primary (e.g. tremor, bradykinesia, rigidity, postural instability, etc.) or secondary (e.g. freezing, micrographia, mask-like expression, unwanted accelerations) motor symptoms [149]. Some of these symptoms are promoted by different pathophysiological neuronal networks and, therefore, may require different biomarkers to detect. Thus, amelioration of a range of PD motor symptoms through least a number of biomarkers need further and future investigations. Viability of biomarker recordings over the course of several years is another concern. The choice of sensing methodology, electrode materials and components in the implants, could potentially increase the viability of biomarker recordings. The use of LFPs, which could be recorded from the same lead used to induce DBS, also offers a more stable biomarker measurements. However, selection of a biomarker that requires a separate recording path from those implanted for DBS may not assure the viability of recordings and require further investigations.

### Towards commercializing closed-loop DBS devices

The promising clinical effects of open-loop DBS have been demonstrated, indicating DBS as a pioneer technology and treatment option to serve neurological patients. Moreover, the significant increase in the number of commercial open-loop DBS devices represents the approach effectiveness. However, like other commercial devices, DBS needs to be automated and modernized. Currently, in the open-loop DBS approach, the stimulation parameters are periodically adjusted by neurologists and the parameters remain constant in the intervals of two subsequent visits regardless of any variations in the patient’s clinical state. Therefore, it may cause brain overstimulation or produce fewer benefits because of insufficient stimulations. Closed-loop DBS can eliminate brain overstimulation and provide maximum stimulation efficiency for those in need of DBS therapy.

Despite the advancements in the closed-loop DBS devices, most current commercial DBS systems operate in an open-loop manner. To date, only one commercial closed-loop DBS device has been manufactured (the RNS system). However, life expectancy of IPGs is not very good for the RNS system. The battery charge is rapidly depleted when stimulation is used heavily, and the control algorithm is currently conventional based on on-demand (On-Off) approach [125]. A closed-loop DBS device requires an adaptive algorithm to learn and optimize the stimulation parameters according to the brain clinical state. Owing to the fact that each patient is different, such an algorithm may need to be patient dependent.

## Conclusion

Recent advances in the closed-loop DBS systems provide a brighter future for patients in need of DBS treatment. The next generation of DBS devices is expected to be fully and automatically programmable, compatible with biomarker variations, and flexible in stimulation type and pattern, to yield greater benefits and fewer side effects. Since a new technology has to be accessible to all patients over the world, the next closed-loop designs need to be inexpensive to become available even in developing countries. In addition, it can be expected that the device operates based on multiple biomarkers to decrease the chance of malfunction and guarantee the robustness of the closed-loop DBS operation. Engineers need to provide a balance and trade-off among the device features by considering the clinical effectiveness, and the technological costs of the device. However, whether the future advancements in the closed-loop DBS devices can result in a better life and efficient care for their users need to be yet demonstrated. Alongside all the advancements and benefits that closed-loop DBS devices can bring to the patients, other brain stimulation methods e.g. optogenetics and ultrasonic stimulations are also receiving attention by research communities.

## Abbreviations

AC: Alternating current
ADC: Analog to digital converter
AP: Action potential
BCI: Brain computer interface
CC: Constant-current-controlled
CMOS: Complementary metal–oxide–semiconductor
CMRR: Common mode rejection ratio
CR: Coordinated-reset
CV: Constant-voltage-controlled
DBS: Deep brain stimulation
DC: Direct current
ECoG: Electrocorticogram
EEG: Electroencephalogram
EMG: Electromyogram
FDA: Food and drug administration
FSCV: Fast-scan cyclic voltammetry
IC: Integrated circuit
LFP: Local field potential
MPTP: 1-methyl-4-phenyl-1,2,3,6-tetrahydropyridine
OCD: Obsessive-compulsive disorder
PD: Parkinson’s disease
PI: Proportional-integral
PID: Proportional-integral-derivative
RNS: Responsive neurostimulator
SCS: Spinal cord stimulation
SoC: System-on-chip
STN: Subthalamic nucleus
VNS: Vagus nerve stimulation
